# Supplementary material for: Interdisciplinary intervention (GAIN) for adults with post-concussion symptoms: a study protocol for a stepped-wedge cluster randomised trial
Source: Trials. 2022 Jul 29;23:613. doi: 10.1186/s13063-022-06572-7 (PMC9338593; doi:10.1186/s13063-022-06572-7)
Supplement: Supplementary file 1 — Additional file 1. Outcome measures. [file 13063_2022_6572_MOESM1_ESM.pdf]

Additional file 1: Outcome measures

	Description	Data source
**Primary outcome**		
**Symptoms** (16 items)	Severity of post-concussion symptoms covering physical, cognitive, and emotional symptoms.	Rivermead Post-Concussion Symptoms Questionnaire (RPQ) [1]
**Secondary outcomes**		
**Participation** (31 items)	Frequency, experienced limitation, and experienced satisfaction in Activities of Daily Living (IADL)	The Utrecht Scale for Evaluation of Rehabilitation-Participation (USER-P) [2]
**Labour market attachment**		
Employment (register based)	Receives no public assistance benefits except from state education fund grants	DREAM Register [3]
Long term sick leave (register based)	Receives public assistance benefits related to illness in more than three consecutive weeks	DREAM Register [3]
Job stability (register based)	Degree of employment (based on whether labor market contributions have been paid)	DREAM Register [3]
Sickness absence (3 items)	Days with sickness absence or days affected by physical or cognitive problems during work/school/ education during the preceding 4 weeks.	Treatment Inventory of Costs in Patients with psychiatric disorders (TiC-P) [4]
Work ability (3 items)	Current workability compared with the best of lifetime	Work Ability Index Short form (WAI-2) [5]
**Consumption of health care**		
General Practitioner (GP) and Psychologists (register based)	Number of contacts to GP and psychologists	National Health Service Registry [6]
Hospitalisations (register based)	Number of days in hospital and number of emergency visits	National patient registry [6]
Medication (3 items)	Use of painkillers, antidepressants, anxiolytics, or hypnotics	Self-constructed
Long term medication (register based)	Persistent use of painkillers, antidepressants, anxiolytics, or hypnotics	Danish National Prescription Registry [6]
Non-pharmacological Treatment and	Use of non-pharmacological treatment and management strategies such as, chiropractor, physiotherapy, psychologist, acupuncture,	Self-constructed

Additional file 1: Outcome measures

management strategies	alternative treatment, exercise, relaxation techniques, bed rest,	
**Quality of life**		
Quality of life (36 items)	Health-related quality of life and mental and physical functioning	Short Form 36 Health Survey (2nd version) (SF-36) [7]
Quality of life (5 items)	Health-related quality of life and mental and physical functioning	EuroQol-5 Domain (EQ-5D) [https://euroqol.org/eq-5d-instruments/]
**Measures of emotional distress, illness perception, and illness behavior**		
Anxiety (6 items)	Health anxiety	Whiteley-6-R [8]
Psychological Distress (8 items)	Screening for Anxiety and depression	SCL-8 [9]
Illness perception (9 items)	Symptom-perpetuating illness perceptions	The Brief Illness Perception Questionnaire (B-IPQ) [10]
Illness behaviours (7 items)	Illness behaviour	The Behavioural Response to Illness Questionnaire (BRIQ) [11]
**Other**		
Subjective improvement (5 items)	General health	5-point Clinical Global Improvement scale (CGI) [12]
Patient satisfaction (10 items)	Satisfaction with the service provided	The Experience of Service Questionnaire (ESQ) [13]
Adverse events (1 item)	Patients self-reported side effects	Self-constructed
Headache	Headache	Self-constructed
Facial perception	Measures perceived changes in the size/ shape of the affected face or head region and other sensory changes (such as feeling of warmth, cold, numbness etc) in the face or head region	Self-constructed from [14, 15]
Interviews	Individual interviews with the following themes: 1) Life with concussion before the intervention, 2) Experiences of the GAIN intervention (main focus), and 3) Life after the intervention	Self-constructed using a semi-structured interview guide. The interviews will be recorded and transcribed.

Additional file 1: Outcome measures

References

1 King NS, Crawford S, Wenden FJ, Moss NEG, Wade DT. The Rivermead post concussion symptoms questionnaire: a measure of symptoms commonly experienced after head injury and its reliability. *J Neurol* 1995;242(9):587–92.

2 Post MWM, Van Der Zee CH, Hennink J, Schafrat CG, Visser-Meily JMA, Van Berlekom SB. Validity of the utrecht scale for evaluation of rehabilitation- participation. *Disabil Rehabil* 2012;34(6):478–85.

3 Hjollund NH, Larsen FB, Andersen JH. Register-based follow-up of social benefits and other transfer payments: Accuracy and degree of completeness in a Danish interdepartmental administrative database compared with a population-based survey. *Scand J Public Health* 2007;35(5):497–502.

4 Kanters TA, Timman R, Zijlstra-Vlasveld MC, Muntingh A, Huijbregts KM, van Steenbergen-Weijenburg KM, et al. Assessing Costs Using the Treatment Inventory Cost in Psychiatric Patients (TIC-P), TIC-P Mini and TIC-P Midi. *J Ment Heal policy Econ* 22(1):15–24.

5 Ebener M, Hasselhorn HM. Validation of short measures of work ability for research and employee surveys. *Int J Environ Res Public Health* 2019;16(18):1–15.

6 Schmidt M, Schmidt SAJ, Adelborg K, Sundbøll J, Laugesen K, Ehrenstein V, et al. The Danish health care system and epidemiological research: From health care contacts to database records. *Clin Epidemiol* 2019;11:563–91.

7 Bjorner JB, Thunedborg K, Kristensen TS, Modvig J, Bech P. The Danish SF-36 Health Survey: Translation and preliminary validity studies. *J Clin Epidemiol* 1998;51(11):991–9.

8 Fergus TA, Kelley LP, Griggs JO. The Whiteley Index–6: An Examination of Measurement Invariance Among Self-Identifying Black, Latino, and White Respondents in Primary Care. *Assessment* 2018;25(2):247–58.

9 Fink P, Ørnbøl E, Huyse FJ, De Jonge P, Lobo A, Herzog T, et al. A brief diagnostic screening instrument for mental disturbances in general medical wards. *J Psychosom Res* 2004;57(1):17–24.

10 Broadbent E, Petrie KJ, Main J, Weinman J. The Brief Illness Perception Questionnaire. *J Psychosom Res* 2006;60(6):631–7.

11 Spence M, Moss-Morris R, Chalder T. The Behavioural Responses to Illness Questionnaire (BRIQ): A new predictive measure of medically unexplained symptoms following acute infection. *Psychol Med* 2005;35(4):583–93.

12 Busner J, Targum SD. The clinical global impressions scale: applying a research tool in clinical practice. *Psychiatry* 4(7):28–37.

13 Barber AJ, Tischler VA, Healy E. Consumer satisfaction and child behaviour problems in child and adolescent mental health services. *J Child Heal Care* 2006;10(1):9–21.

14 Dagsdóttir LK, Skyt I, Vase L, Baad-Hansen L, Castrillon E, Svensson P. Reports of perceptual distortion of the face are common in patients with different types of chronic oro-facial pain. J Oral Rehabil. 2016 Jun;43(6):409-16.

15 Skyt I, Dagsdóttir L, Vase L, Baad-Hansen L, Castrillon E, Roepstorff A, et al. Painful stimulation and transient

blocking of nerve transduction due to local anesthesia evoke perceptual distortions of the face in healthy volunteers. J Pain. 2015 Apr;16(4):335-45.
