# Supplementary figures and images for: Interdisciplinary intervention (GAIN) for adults with post-concussion symptoms: a study protocol for a stepped-wedge cluster randomised trial
Source: Trials. 2022 Jul 29;23:613. doi: 10.1186/s13063-022-06572-7 (PMC9338593; doi:10.1186/s13063-022-06572-7)

Additional file 2: Organisation

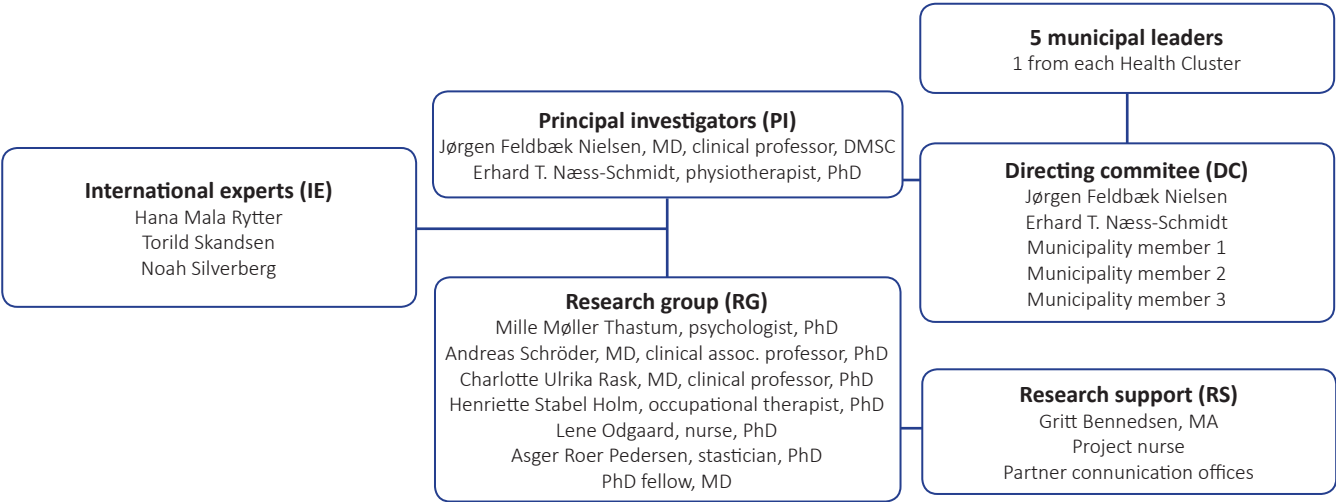

Supplement: Supplementary file 2 — Additional file 2. Organisation. [file 13063_2022_6572_MOESM2_ESM.pdf]
