# Supplementary material for: Interdisciplinary intervention (GAIN) for adults with post-concussion symptoms: a study protocol for a stepped-wedge cluster randomised trial
Source: Trials. 2022 Jul 29;23:613. doi: 10.1186/s13063-022-06572-7 (PMC9338593; doi:10.1186/s13063-022-06572-7)
Supplement: Supplementary file 3 — Additional file 3. [file 13063_2022_6572_MOESM3_ESM.pdf]

Date 19-04-2021
Case manager Helle Nikkel
komite@rm.dk
Tel. + 4578410186
Case no. 1-10-72-330-20

Jørgen Feldbæk Nielsen, Professor, chief physician, MD
Hammel Neurorehabilitation Centre and University Research Clinic
Voldbyvej 15
8450 Hammel

Regionshuset Viborg
Regional Secretariat
Legal Office
The Central Denmark Region Committees on Health Research Ethics
Skottenborg 26 DK-8800 Viborg
Tel. +45 7841 0183
komite@rm.dk
www.komite.rm.dkDate

Final approval

Project: Move on after concussion 2.0. Interdisciplinary intervention for patients with post-concussion symptoms 3-6 months after injury (GAIN 2.0)

The Central Denmark Region Committees on Health Research Ethics, Committee I, confirm the receipt of an e-mail dated 19 April 2021 in response to the committee's decision of 16 April 2021, which set out the conditions for the approval of the project.

Decision:
The decision was made in accordance with Executive Order no. 1338 of 1 September 2020 on the ethical treatment of health science research projects and health data science research projects.
The conditions of the approval are considered fulfilled. The project is hereby finally approved.
The approval applies to the notified test sites and the notified test leader in Denmark.

The approval is valid until 31 December 2023 and includes the following documents:
- Experimental protocol, version 6, dated 19 April 2021.
- Pamphlet on recruitment, version and date not specified, file named version 1, dated December 7, 2020.
- Recruitment material, dated 17 March 2021.
- Participant information, dated March 29, 2021, file named version 4, dated March 29, 2021.
- Declaration of consent, version 3, dated 17 March 2021.
- Questionnaires submitted on 16 December 2020 have been approved for distribution.

It is noted that the committee is not a competent authority regarding the rules on data protection, and that the committee assumes that the content of the project regarding this is in accordance with Regulation No 2016/679 of the European Parliament and of the Council of 27 April 2016 on the protection of individuals with regard to the processing of personal data and on the free movement of such data and the Data Protection Act.
Initiation of the project against the approval can be punished with a fine or imprisonment, cf. § 41. of the Scientific Ethical Committees Act.

Changes:
If significant changes are made to the protocol during the implementation of the project, these must be notified to the committee in the form of additional protocols. The changes may only be implemented after approval by the committee, cf. the Scientific Ethical Committees Act, § 27, subsection 1.
Notification of additional protocols must be done electronically at www.drvk.dk with the already assigned notification number and password. Significant changes include changes that may affect the safety of the subjects, interpretation of the scientific evidence on which the project is based and the implementation or management of the project. These can be, for example, changes in inclusion and exclusion criteria, trial design, number of subjects, project extension, trial procedures, treatment duration, effect parameters, changes in the trial supervisors or trial sites and substantive changes in the written information material for the subjects. Where new information means that the researcher is considering changing the procedure or stopping the experiment, the committee should be informed.

Side effects and incidents:
Ongoing reporting The committee must be notified immediately if any serious, unexpected side effects or serious incidents occur during the project, cf. of the Scientific Ethical Committees Act § 30 (1).
The report must be accompanied by comments on any consequences for the trial. Only side effects and events that have occurred in Denmark should be reported. Notification must be made no later than 7 days after the sponsor or the person responsible for the trial has become aware of the case. When reporting, a form can be used, which can be found at www.nvk.dk. The form with any documents must be submitted electronically in pdf format to komite@rm.dk

Annual report Once a year throughout the trial period, the committee must have sent a list of all presumed serious (expected and unexpected) side effects and serious incidents that occurred during the trial period together with a report on the safety of the subjects, cf. the Scientific Ethical Committees Act § 30, 2. If there have been no serious side effects and events, this must also be reported.
When reporting, a form can be used, which can be found at www.nvk.dk. The form with any documents must be submitted electronically in pdf format to komite@rm.dk

Termination:
The person responsible for the experiment must notify the committee of this within 90 days after the end of the project, cf. Scientific Ethical Committees Act § 31 (1). The project is considered

completed when data collection is completed. If the project is interrupted earlier than planned, a justification for this must be sent to the committee no later than 15 days after the decision has been made, cf. Scientific Ethical Committees Act § 31 (2).

If the project is not started, this and the reason for this must be notified to the committee. The committee requests a copy of the final research report or publication, cf. Scientific Ethical Committees Act § 28 (2). In this connection, we must draw attention to the fact that there is an obligation to publish both negative, positive and inconclusive experimental results, cf. Scientific Ethical Committees Act § 20 (1), No. 8.

Supervision:
The committee supervises that the project is carried out in accordance with the approval, cf. . Scientific Ethical Committees Act § 28 and § 29.

Sincerely
Helle Nickel
Secretary

Copy to: RN, PhD, Lene Odgaard, Hammel Neurorehabilitation Centre and University Research Clinic
