# Supplementary material for: Interdisciplinary intervention (GAIN) for adults with post-concussion symptoms: a study protocol for a stepped-wedge cluster randomised trial
Source: Trials. 2022 Jul 29;23:613. doi: 10.1186/s13063-022-06572-7 (PMC9338593; doi:10.1186/s13063-022-06572-7)
Supplement: Supplementary file 4 — Additional file 4. [file 13063_2022_6572_MOESM4_ESM.pdf]

Regionshuset
Viborg

Regionssekretariatet
Juridisk kontor
De Videnskabsetiske Komitéer
For Region Midtjylland

Skottenborg 26
DK-8800 Viborg
Tel. +45 7841 0183
komite@rm.dk
www.komite.rm.dk

Jørgen Feldbæk Nielsen, Professor, overlæge, dr.med.
Regionshospitalet Hammel Neurocenter
Voldbyvej 15
8450 Hammel

Dato 19-04-2021
Sagsbehandler Helle Nikkel
komite@rm.dk
Tel. +4578410186
Sagsnr. 1-10-72-330-20

Endelig godkendelse

Projekt: Videre efter hjernerystelse 2.0. Interdisciplinary intervention for patients with post-concussion symptoms 3-6 months post-injury (GAIN 2.0)

De Videnskabsetiske Komitéer for Region Midtjylland, Komité I, bekræfter modtagelsen af mail af 19. april 2021 som svar på komitéens afgørelse af 16. april 2021, hvori der opstilledes betingelser for godkendelsen af projektet.

Afgørelse:

Afgørelsen er truffet efter lovbekendtgørelse nr. 1338 af 1. september 2020 om videnskabsetisk behandling af sundhedsvidenskabelige forskningsprojekter og sundhedsdatavidenskabelige forskningsprojekter.

Betingelserne for godkendelsen anses for opfyldt. Projektet er hermed endeligt godkendt.

Godkendelsen gælder for de anmeldte forsøgssteder og den anmeldte forsøgsansvarlige i Danmark.

Godkendelsen gælder til den 31. december 2023 og omfatter følgende dokumenter:

- Forsøgsprotokol, version 6, dateret 19. april 2021.
- Pjece mhp. rekruttering, version og dato ikke angivet, fil navngivet version 1, dateret 7. december 2020.
- Rekrutteringsmateriale, dateret 17. marts 2021.

* Deltagerinformation, dateret 29. marts 2021, fil navngivet version 4, dateret 29. marts 2021.
* Samtykkeerklæring, version 3, dateret 17. marts 2021.
* Spørgeskemaer indsendt den 16. december 2020 er godkendt til udlevering.

Det bemærkes, at komitéen ikke er ressortmyndighed vedr. regelsættet om databeskyttelse, og at komiteen forudsætter, at projektets indhold vedr. dette er i overensstemmelse med Europa-Parlamentets og Rådets forordning nr. 2016/679 af 27. april 2016 om beskyttelse af fysiske personer i forbindelse med behandling af personoplysninger og om fri udveksling af sådanne oplysninger og databeskyttelsesloven.

Iværksættelse af projektet i strid med godkendelsen kan straffes med bøde eller fængsel, jf. komitélovens § 41.

Ændringer:

Foretages der væsentlige ændringer i protokolmaterialet under gennemførelsen af projektet, skal disse anmeldes til komitéen i form af tillægsprotokoller. Ændringerne må først iværksættes efter godkendelse fra komitéen, jf. komitélovens § 27, stk. 1.

Anmeldelse af tillægsprotokoller skal ske elektronisk på *www.drvk.dk* med det allerede tildelte anmeldelsesnummer og adgangskode.

Væsentlige ændringer er bl.a. ændringer, der kan få betydning for forsøgspersonernes sikkerhed, fortolkning af den videnskabelige dokumentation, som projektet bygger på samt gennemførelsen eller ledelsen af projektet. Det kan fx være ændringer i in- og eksklusionskriterier, forsøgsdesign, antal forsøgspersoner, projektforlængelse, forsøgsprocedurer, behandlingsvarighed, effektparametre, ændringer om de forsøgsansvarlige eller forsøgssteder samt indholdsmæssige ændringer i det skriftlige informationsmateriale til forsøgspersonerne.

Hvor nye oplysninger betyder, at forskeren overvejer at ændre proceduren eller stoppe forsøget, skal komitéen orienteres om det.

Bivirkninger og hændelser:

Løbende indberetning
Komitéen skal omgående underrettes, hvis der under projektet optræder formodet alvorlige, uventede bivirkninger eller alvorlige hændelser, jf. komitélovens § 30, stk. 1.

Indberetningen skal ledsages af kommentarer om eventuelle konsekvenser for forsøget. Det er kun bivirkninger og hændelser forekommet i Danmark, der skal indberettes. Underretning skal ske senest 7 dage efter, at sponsor eller den forsøgsansvarlige har fået kendskab til tilfældet.

Ved indberetning kan anvendes et skema, der findes på *www.nvk.dk*. Skemaet med evt. bilag skal indsendes elektronisk i pdf-format til *komite@rm.dk*.

Årlig indberetning
Én gang årligt i hele forsøgsperioden skal komitéen have tilsendt en liste over alle formodet alvorlige (ventede og uventede) bivirkninger og alvorlige hændelser, som er indtruffet i forsøgsperioden sammen med en rapport om forsøgspersonernes sikkerhed, jf. komitélovens § 30, stk. 2. Har der ikke været alvorlige bivirkninger og hændelser skal dette ligeledes indberettes.

Ved indberetning kan anvendes et skema, der findes på *www.nvk.dk*. Skemaet med evt. bilag skal indsendes elektronisk i pdf-format til *komite@rm.dk*.

Afslutning:

Den forsøgsansvarlige skal senest 90 dage efter afslutningen af projektet underrette komitéen herom, jf. komitélovens § 31, stk. 1. Projektet regnes som afsluttet, når indsamling af data er afsluttet.

Afbrydes projektet tidligere end planlagt, skal en begrundelse herfor sendes til komitéen senest 15 dage efter, at beslutningen er truffet, jf. komitélovens § 31, stk. 2.

Hvis projektet ikke påbegyndes, skal dette samt årsagen hertil meddeles komitéen.

Komitéen beder om kopi af den afsluttende forskningsrapport eller publikation, jf. komitélovens § 28, stk. 2. Vi skal i den forbindelse gøre opmærksom på, at der er pligt til at offentliggøre både negative, positive og inkonklusive forsøgsresultater, jf. komitélovens § 20, stk. 1, nr. 8.

Tilsyn:

Komitéen fører tilsyn med, at projektet udføres i overensstemmelse med godkendelsen, jf. komitélovens § 28 og § 29.

Venlig hilsen

Helle Nikkel

Helle Nikkel
Sekretær

Kopi til:

- Sygeplejerske, ph.d., Lene Odgaard, Regionshospitalet Hammel Neurocenter
