# Supplementary material for: Interdisciplinary intervention (GAIN) for adults with post-concussion symptoms: a study protocol for a stepped-wedge cluster randomised trial
Source: Trials. 2022 Jul 29;23:613. doi: 10.1186/s13063-022-06572-7 (PMC9338593; doi:10.1186/s13063-022-06572-7)
Supplement: Supplementary file 5 — Additional file 5. [file 13063_2022_6572_MOESM5_ESM.pdf]

Informeret samtykke til deltagelse i interviewundersøgelse

Forskningsprojektets titel: Interviewundersøgelse af oplevelser af nyt vejlednings- og træningsforløb for langvarige følger efter hjernerystelse ("Videre efter hjernerystelse" (GAIN 2.0))

Erklæring fra deltageren.

Jeg har fået skriftlig og mundtlig information og jeg ved nok om formål, metode, fordele og ulemper til at sige ja til at deltage i interviewet om mine oplevelser af vejlednings- og træningsforløbet, ”Videre efter hjernerystelse” (GAIN 2.0).

Jeg ved, at det er frivilligt at deltage i interviewet, og at jeg altid kan trække mit samtykke tilbage uden at miste mine nuværende eller fremtidige rettigheder til behandling.

Jeg giver samtykke til, at deltage i interviewet, og har fået en kopi af dette samtykkeark samt en kopi af den skriftlige information om projektet til eget brug.

Den interviewedes navn: ____

Dato: ______________ Underskrift: ____

Ønsker du at blive informeret om forskningsprojektets resultat

Ja _____ (sæt x) Nej _____ (sæt x)

Erklæring fra den, der afgiver information:

Jeg erklærer, at den interviewede har modtaget mundtlig og skriftlig information om interviewet. Efter min overbevisning er der givet tilstrækkelig information til, at der kan træffes beslutning om deltagelse i interviewet.

Navnet på den, der har afgivet information:

Dato: ______________ Underskrift: ____
