# Supplementary material for: Interdisciplinary intervention (GAIN) for adults with post-concussion symptoms: a study protocol for a stepped-wedge cluster randomised trial
Source: Trials. 2022 Jul 29;23:613. doi: 10.1186/s13063-022-06572-7 (PMC9338593; doi:10.1186/s13063-022-06572-7)
Supplement: Supplementary file 6 — Additional file 6. Samtykke GAIN 2.0. [file 13063_2022_6572_MOESM6_ESM.pdf]

Samtykke GAIN 2.0

Deltagerinformation GAIN 2.0

[Attachment: "Skriftlig deltagerinformation.pdf"]

Dato for mundtlig information om projektet "Videre efter hjernerystelse" ________________________

Navn på person, der giver mundtlig information om projektet "Videre efter hjernerystelse" ________________________

Jeg [info_person_gain] erklærer, at forsøgspersonen har modtaget mundtlig og skriftlig information om projektet "Videre efter hjernerystelse".
Efter min overbevisning er der givet tilstrækkelig information til, at der kan træffes beslutning om deltagelse i projektet. ______________________________

Dato for samtykke til deltagelse i projektet "Videre efter hjernerystelse" ________________________

Jeg [id_arm_1][fornavne] [id_1][efternavn] har modtaget information om projektet "Videre efter hjernerystelse" både mundtligt og skriftligt og jeg ved nok om formål, metode, fordele og ulemper til at sige ja til at deltage. ______________________________
Jeg ved, at det er frivilligt at deltage, og at jeg altid kan gå ud af projektet, uden at det vil påvirke min nuværende eller fremtidige behandling.
Jeg giver hermed samtykke til at deltage i forskningsprojektet.

Upload samtykke (i fald ej underskrevet digitalt)
